# Perceived quality of padel users: adaptation and validation of the QPadel assessment tool

**DOI:** 10.3389/fspor.2024.1526512

**Published:** 2024-12-20

**Authors:** Nicolás Fernández Martínez, Pablo Gálvez-Ruiz, Ramón Gómez Chacón, Alejandro Lara-Bocanegra

**Affiliations:** ^1^ Cardenal Spínola CEU University Studies Center, Sevilla, Spain; ^2^ Faculty of Social Sciences and Law, Valencian International University, Valencia, Spain; ^3^ Universidad Loyola Andalucía, Seville, Spain

**Keywords:** padel, perceived quality, sport management, sports services, sports facilities

## Abstract

**Introduction:**

Padel is currently an emerging sport that has experienced significant growth, enjoying popularity and widespread accessibility among the population. However, the padel context lacks a tool to assess the perceived quality of users in padel facilities and sports services. This study aims to adapt and validate an evaluation tool based on a literature review.

**Methods:**

The sample included 402 users (298 men and 104 women, predominantly a frequency of play of 1–2 days a week for 1–2 h) from clubs across the Andalusian Autonomous Community (South of Spain). Psychometric properties were evaluated using exploratory and confirmatory factor analysis, internal consistency through Cronbach's Alpha indicator and composite reliability, as well as convergent and discriminant validity, using the statistical software SPSS (v.22).

**Results:**

The findings demonstrated adequate psychometric properties in various analyses (exploratory and confirmatory factor analysis), showing both internal consistency and validity (convergent and discriminant).

**Discussion:**

The QPadel tool represents a significant contribution and advancement in the academic literature, with potential positive impacts on decision-making for improving padel facilities and services, as well as enhancing competitiveness.

## 1 Introduction

Padel stands out as one of the emerging sports, experiencing unprecedented growth in recent years, both in terms of participants and the number of facilities. Its popularity is so high that the number of padel players surpasses that of tennis players in countries like Spain, Sweden, and Portugal. Notably, there has been an increase in participation, international expansion of padel infrastructure, and its growing economic importance (1).

The consolidation of padel as a sport of growing popularity and accessibility in various social contexts can be attributed to multiple factors that favour its practice and dissemination: easy to learn, suitable for different ages, gender, abilities or physical condition, or economic access to practice facilitating its democratization. In addition, it encourages the friendship or peer group factor, satisfaction or fun (2), social engagement and interaction among participants, promoting values of cooperation and mutual respect. The combination of these factors contributes significantly to the rise and consolidation of padel in today's sporting landscape (3).

The increase in the number of people interested in padel comes both from the players themselves and from those attending sporting events related to the sport (4). In Spain, López (5) indicated that it is the sport with the greatest development in the last 23 years with a growth of 1,947.41%, more specifically 20% in the last 10 years according Courel-Ibáñez et al. (6), being also the country with the highest number of padel clubs between 2019 and 2021 with a growth of 11%, experiencing the highest Google search for the word padel (1). This situation is transferred to the evolution of the number of licences, whose evolution since 2012 has shown a permanent growth experiencing its hatching in the post-pandemic period thanks to the fact that its practice takes place, mostly, in outdoor facilities. It has gone from 75 thousand licenses in 2020 to over 90 thousand in 2021 (7). In terms of padel facilities, Spain is the country with the highest number of courts in the world (15,300), with 279 million euros spent on the construction of courts in the last two years (1). All these data indicate that padel is one of the most emerging and fastest-growing sports of the 21st century (8).

The evolution experienced in the practice of padel and its economic impact are indicative of the importance of adapting and continuously improving the quality of the services offered. In fact, this impact of padel has focused the attention of the scientific community with recent studies aimed at analysing variables that affect the game and/or players,. however there is a significant lack in the academic literature of studies oriented to the evaluation of the perception of padel users or players on quality, despite its extensive analysis in a variety of sports organisations as a result of the importance of its study as a starting point to achieve satisfaction and future behavioural intention.

Service quality starts from the satisfaction of expectations, i.e., from the comparison of expectations with the service they perceive they have received (9, 10), and has been a widely analysed construct in the sport industry and specifically in sport services [e.g., (11–14)]. In the context of padel and in the face of the latent emerging demand for associated sport services, it is not an easy task to provide satisfactory and high quality services to all customers (15). In this way, knowing which attributes padel users consider most relevant is decisive for an adequate quality management within the service provision process. In this sense, the validation of quality assessment tools specific to padel becomes a critical resource for measuring and improving the user experience (16).

However, there has been an increase in scientific research whose main focus is on areas such as performance analysis, psychology and physiology, while sport management occupies a less prominent place in the literature as reflected in the systematic review by Sánchez-Alcaraz et al. (17). Specifically, research oriented towards sport management aims to analyse the evolution of federal licences (3, 8), an analysis of the World Padel Tour in terms of expenditure per attendee (18) and the cost-benefit of its organisation (19), the standardisation of facilities (20), the viability of padel centres for the development of business plans (21), and a study on user satisfaction (22), which is the most relevant to the present research, involving two padel clubs and a sample of 36 participants. Therefore, we consider that there is a notable lack of research aimed at assessing the perception of padel users, and in parallel this situation suggests a potential opportunity for the validation of instruments that allow the assessment of constructs as relevant as perceived quality in this specific context. In this way, achieving high levels of quality in service provision is considered one of the indispensable requirements when it comes to obtaining adequate competitiveness and viability in organisations, and for because of this it is necessary to pay attention to all the components surrounding the service in order to achieve the greatest possible homogeneity in them (23).

In this context, perceived quality in sport services emerges as a fundamental concept within the interaction that takes place between organisations and their users, being fundamental for the success of organisations and the loyalty of their customers (24), as well as for the increase of competitiveness (25). Within this relationship, the theory of perceived quality suggests that consumers evaluate the quality of a service based on the discrepancy between their prior expectations (or expected service) and their actual perception of the service received (the way it has been performed) (9, 26, 27). Based on this, the two main models are founded on a technical or result dimension and a functional or process dimension (9). On the other hand, series of attributes or dimensions such as tangible elements, reliability, responsiveness, safety and empathy (27), give rise in this second case to one of the most widely used tools (SERVQUAL). This tool has been reconfigured, tested and modified to be useful for measuring customer perceptions in a variety of industries (28) despite the various criticisms received for its lack of specificity or universal applicability (29–32) and even for psychometric aspects (33). This is why the best system is one that is generated or adapted to each organisation according to its characteristics and needs (23).

Perceived quality is currently still the subject of research in various sectors. Specifically in the sport industry and especially in sport services, understanding the relationship of service quality and customer experience to be crucial to improve customer loyalty and in turn build long-term relationships (34), with previous studies revealing that service quality improves both customer satisfaction and loyalty (13, 35, 36), as does experiential quality (37). In addition to the numerous studies using the SERVQUAL scale by Parasuraman et al. (27), other tools available for the evaluation of sport services are the QUESC scale (38), the SQFS scale (39), the SQAS scale (40) and its reduced version SQAS-19 (28), the SQS-FC scale (41), the QSCSEF scale (42), the CALIDFIT scale (43) and the CECASDEP scale and its reduced version (12, 44), among others. There are also validated tools for other contexts, such as the SSQRS scale for recreational sports (45), the EVENTQUAL scales (46) or Eventserv (47) and its reduced version Eventserv-Short (48) for spectators of sporting events. Participants also receive attention in different contexts such as fantasy sports websites (49), running races (50), duathlon (51), or open water swimming (52).

In the specific context of padel, the study by Aparicio-Sarmiento et al. (22) used the EPOD scale (53) keeping the scale intact with 28 items distributed in 4 dimensions (sports technicians, material resources, activities, and image of the organisation), a tool that was subsequently validated in a sample of athletes participating in physical activities in a multi-sport centre using a 29-item version (54). In the study by Aparicio-Sarmiento et al. (22), although they reported adequate internal consistency (*α* = 0.92), this is a value corresponding to the global scale and no information is provided on the psychometric properties of the tool applied to this specific context (exploratory and confirmatory factor analysis). Therefore, we consider it necessary to explore new options for the assessment of perceived quality in this context since, as stated by Haensel and Hoffmann (55), the dimensions of service quality can be significantly different depending on the type of business.

Based on different reviews in the scientific literature (40, 56–59) and extending it, Table 1 presents the main dimensions of a total of 34 studies that use different tools or adaptations for the evaluation of perceived quality in different sport services. The use of the dimensions “staff” and “programmes” are the most frequent in the literature, forming part of 33 and 25 scales, respectively. In the case of “facilities”, a dimension present in 28 scales, there are different terminologies to refer to the infrastructure that enables sport practice, although certain scales use what we could call a second level when referring to “sport spaces”, understanding these as the specific enclosures provided with the necessary means that allow practice (including here other uses such as learning or training and competition). In this sense, the scales CECASDEP, CECASDEP-R, SQAS and the adaptation of SQAS establish a differentiation between the physical installation and the specific facilities or spaces for carrying out an activity or exercise (e.g., gymnasium, swimming pool, or in the case of the present research, padel court), while in the case of the EPOD2 scale, the 3 items of the so-called “spaces” dimension refer to changing rooms (2 items) and cleanliness (1 item). Material” or “equipment” is another dimension frequently used in different scales, using some of these terms to refer to the elements that allow the adequate development of the contents during sport practice. Finally, other dimensions used in some scales have been included, such as tangible elements, cleanliness, changing rooms, user care, or information and communication, among others.

**Table 1:** Scales for assessing the perceived quality of sport services.

Model name, authors and year	Main dimensions					Other dimensions to consider
	Programme	Personnel	Facilities	Material or equipment	Specific sports venues	
SAFS (Chelladurai et al.) (60)	Yes	Yes	Yes			
QUESC (Kim and Kim) (38)	Yes	Yes	Physical environment			Ambiance, information available
CERM CSQ (Howat et al.) (61)	Yes	Yes	Yes			Secondary services
Han (62)	Yes	Yes	Yes			Public relations, cost
FITSSQ (Papadimitriou and Karteroliotis) (63)	Yes	Yes	Yes			Other services
Brady and Cronin (64)	Yes	Yes	Yes			
AQUASERV (López) (65)		Yes	Yes			
SQFS (Chang and Chelladurai) (39)	Yes	Yes	Physical environment			
Costa et al. (66)	Yes	Yes	Yes			Tangibles, security
Alexandris et al. (67)		Yes	Yes			
SQAS (Lam et al.) (40)	Yes	Yes	Yes	Yes	Yes	Locker rooms
SSQRS (Ko and Pastore) (45)	Yes	Yes	Environment			
QUESC (Afthinos et al.)—ad (68)	Yes	Yes				Ambiance, information available
Sanz et al. (69)	Yes	Yes	Yes	Yes		
HAFSQ (Dhurup et al. (70)	Yes	Yes	Attractiveness	Yes		Functionality, accessibility, ambience, security
Langrosen and Langrosen (71)		Yes				
SFC-PSQS (Uçan) (72)		Yes	Yes			
NEPTUNO (Calabuig et al. (73)	Yes		Environment	Yes		Cleanliness
QSPORT-10 (Rial et al. (74)		Yes	Yes			
SERVPERF (Hwanleep et al.)—ad (75)		Yes	Physical Factor			
EPOD (Nuviala et al.) (54)	Yes	Yes		Yes		Image
SQS-FC (Yildiz) (41)	Yes	Yes	Physical environment			
QSPORT-14 (Yildiz and Kara (76)	Yes	Yes	Yes			
EPOD2 (Nuviala et al.) (77)	Yes	Yes		Yes	Yes	Communication, technical aspects
CALIDFIT (García-Fernández et al.) (43)	Yes	Yes	Yes			
Jasinkas et al.—ad SERVQUAL, SQAS, QUESC (78)	Yes	Yes		Yes		
QGOLF-9 (Serrano-Gómez et al.) (79)		Yes	Yes			
SQAS (Yu et al.)—ad (80)	Yes	Yes	Yes	Yes	Yes	Locker rooms, nursery
CECASDEP (Gálvez-Ruiz and Morales-Sánchez) (12)	Yes	Yes	Yes		Yes	Locker rooms, user services
CECASDEP-R (Gálvez-Ruiz et al.) (44)	Yes	Yes	Yes		Yes	Locker rooms, user services
SQAS-19 (Walker et al.) (28)	Yes	Yes	Yes		Yes	
Maksimović et al. (81); Tsitkari et al. (82)—ad Brady and Cronin (64) adjusted by Alexandris et al. (67)		Yes	Yes			
QIF-AG (Campos et al.) (83)		Yes				
Montero-Vieira and Ferrera (84)	Yes	Yes	Yes			

Note: ad, adapted version.

Therefore, the objective of this work is focused on adapting a perceived quality assessment tool to the padel context, analyzing the psychometric properties necessary for its validation using a sample of users of sports facilities and services of padel, thus responding to the gap existing in the academic literature in a specific context of great relevance today.

## 2 Methodology

### 2.1 Participants

The sample consisted of a total of 402 padel users (298 men and 104 women), all from the Autonomous Community of Andalusia. In relation to the characteristics of the sample, the age range of the participants was between 18 and 68 years old, with the highest percentages concentrated in the 19 and 21 age group (9.2%, 9.0% and 12.2%, respectively), 63.7% of the participants indicated that they were not members of a padel club, 73.4% practised padel in a private facility, while in terms of occupation, percentages close to 50% were obtained for both students (48%) and workers (52%). In relation to proximity, 77.1% indicated that it took them less than 15 min to get to the sports facility, and in relation to the practice profile, 61.4% played padel between 1 and 2 times a week, with the majority using between 1 and 2 h as playing time per day (89.6%) (Table 2).

**Table 2:** Profile and socio-demographic characteristics of the participants.

Variables	N (%)	Variables	N (%)
Gender		Arrival time	
Male	298 (74.1)	<15 min	310 (77.1)
Female	104 (25.9)	15–25 min	84 (20.9)
Occupation		>25 min	8 (2.0)
Student	193 (48.0)	Weekly practice (days)	
Worker	209 (52.0)	1–2	247 (61.4)
Level of education		3–4	130 (32.3)
Secondary education	37 (9.2)	5–6	21 (5.2)
High school	58 (14.4)	All 7 days	4 (1.0)
Vocational training	104 (25.9)	Hours of practice/day	
Others	3 (0.7)	1–2	360 (89.6)
Without studies	2 (0.5)	3–4	31 (7.7)
Type of facility		5–6	7 (1.7)
Public	84 (20.9)	All 7 days	4 (1.0)
Private	295 (73.4)	Monthly expenditure	
Gym	16 (4.0)	<15€	97 (24.1)
Other	7 (1.7)	15–30€	95 (23.6)
Padel club member		31–60€	99 (24.6)
Yes	146 (36.3)	61–90€	52 (12.9)
No	256 (63.7)	91–120€	32 (8.0)
		>120€	27 (6.7)

### 2.2 Instrument

The tool used in this study is adapted from a previous research work developed in the context of sports services (12), and has been adapted by research carried out in Mexico (85), Chile (86), Ecuador and Colombia (87) in the context of sports services, obtaining adequate psychometric properties.

For the adaptation to the context of padel, a committee of 4 experts of the Degree in Physical Activity and Sport Sciences from different universities was created, of which 2 were specialists in racket sports and sports management, 1 specialist in sports management and owner of a padel club, and 1 specialist in methodology, all with 15 years of professional experience. The Delphi methodology (88, 89) was used to configure the committee of experts, guaranteeing anonymity at all times between the participating members of the committee. The process had 2 rounds of review, analyzing the relevance of the items and making the necessary. Thus, the name of 3 dimensions of the original tool was modified, specifically padel courts instead of sports spaces, padel activity programme instead of activity programme, and padel technicians instead of teacher-monitor. On the other hand, 1 item of the padel courts dimension was eliminated as it lacked possible adaptation (“the acoustics of the sports spaces are good”), and the content of some items was slightly adapted to be specifically oriented to the context of padel (e.g., “the external dimensions of the courts where I play are adequate” instead of “the dimensions of the space where the activity takes place are adequate”, or “the courts offer me safety” instead of “the sports space offers safety”).

Thus, the version used for the present research was a model composed of forty-eight items maintaining the five-point response format (1 = “I do not agree at all” to 5 = “I strongly agree”) and the five original dimensions: sports facility (ID, 10 items), padel courts (PP, 9 items), changing rooms (V, 12 items), padel activities programme (PAP, 9 items), and padel technicians (TP, 8 items). A specific block was included with the following socio-demographic questions: age, gender, level of studies, professional situation, type of club where padel is played, time to get to the padel facility, years of practice, days of practice per week, hours of practice per day and amount of money spent per month to practice padel.

### 2.3 Procedure

The Andalusian Padel Federation was contacted and agreed to participate in the research by providing the link to access the tool through their website. Data were collected over a 12-month period, specifically between 23 April 2022 and 23 April 2023. The questionnaire was administered in an online format created with Google forms, with all questions set to mandatory to eliminate non-response bias. The wording requested voluntary participation and guaranteed the anonymity of the responses, followed by the obligation to mark informed consent in order to continue with the process. The questionnaire took approximately 8–10 min to complete.

### 2.4 Data analysis

Descriptive statistics (means and standard deviation) and the normality of the data were calculated using univariate skewness and kurtosis values. To check the internal structure of the questionnaire adapted to the context of padel, an exploratory factor analysis (EFA), principal component analysis and oblimin oblique rotation were carried out, checking the relevance through Bartlett's test of sphericity and the Kaiser-Meyer-Olkin test (KMO), in addition to the percentage of variance explained. Measures to check the quality of the results were communalities [≥0.50; (90)] and factor weights [≥0.50; (91)]. The internal consistency of the dimensions was assessed using Cronbach's Alpha [α≥; (92)]. Several measures were used to assess model fit quality in confirmatory factor analysis (CFA): (1) *χ*^2^ and its differences of degrees of freedom [*χ*^2^/df ≤ 3; (93)], the comparative fit index (CFI), the incremental fit index (IFI), the Tucker–Lewis index (TLI), parsimony comparative fit index (PCFI) and root mean square error of approximation (RMSEA). According to Geiser (94), a model with a good fit to the data is characterised by CFI, IFI and TLI values above 0.90 and RMSEA values of 0.08 or lower, while values ≥0.60 are adequate for the PCFI indicator (63). Additionally, composite reliability [CR > 0.70; (95)], average variance extracted [AVE > 0.50; (95)], convergent and discriminant validity were calculated.

## 3 Results

Normality test results showed adequate skewness and kurtosis values for all variables, falling within the conventional criteria for normality [±3; (96)]. The mean value of the dimensions showed a very low difference (range of 0.30), namely between 3.96 for the dimensions sports facility (SD = 0.34) and changing room (SD = 0.96) and 4.26 for the dimension padel technicians (SD = 0.92). Within this first phase of analysis, the internal consistency of the dimensions was checked by means of Cronbach's Alpha indicator, obtaining in all cases values above the recommended 0.90.

The relevance of the EFA provided satisfactory results for both Bartlett's test of specificity [*χ*^2^ (1128) = 16103.81; *p* < 0.001] and the KMO test (0.956), thus showing an adequate factor structure for the different dimensions that explains 69.70% of the variance. The EFA results showed communalities above 50% for all items except for items ID4 (0.45) and ID5 (0.47), and factor loadings for the items above 0.50. The factor structure of the EFA was assessed by means of a CFA in order to test the model fit, using a maximum likelihood estimation method to determine how the items represented the different constructs. The results obtained showed a satisfactory fit according to the different indices considered, with a *χ*^2^/df value below 3 (2.17), CFI (0.92), IFI (0.92) and TLI (0.91) values above the minimum cut-off point, the PCFI indicator (0.86) above 0.60 and the RMSEA index [0.054 (LO = 0.051; HI = 0.057)] below the 0.08 cut-off point. In the case of the factor loadings between observable variables, they showed high values, above 0.60 in all cases, without the need to eliminate any item, as good results were obtained in all cases in all the analyses carried out to check the psychometric properties (Table 3).

**Table 3:** Properties of the items of QPadel.

Construct and items	M	SD	Sk/Ku	λ (EFA)	λ (CFA)
ID: Sports Facility (*α* = 0.95; CR = 0.94; AVE = 0.59)					
ID1	4.02	1.09	0.66/0.97	0.61	0.67
ID2	3.61	1.06	0.09/1.22	0.64	0.78
ID3	3.91	1.15	0.51/1.23	0.62	0.70
ID4	3.95	1.20	0.55/1.32	0.56	0.77
ID5	4.06	0.81	0.10/1.49	0.54	0.79
ID6	3.98	0.82	0.03/1.50	0.51	0.73
ID7	4.06	0.83	0.12/1.54	0.62	0.82
ID8	4.06	0.81	0.12/1.48	0.64	0.82
ID9	4.01	0.83	0.03/1.54	0.57	0.83
ID10	3.98	0.81	0.03/1.49	0.53	0.77
PP: Padel Courts (*α* = 0.92; CR = 0.92; AVE = 0.57)					
PP1	4.16	1.02	1.08/0.46	0.59	0.63
PP2	4.16	0.95	1.05/0.68	0.59	0.70
PP3	3.92	1.04	0.74/0.07	0.68	0.81
PP4	4.10	0.98	0.81/0.23	0.63	0.78
PP5	4.18	0.98	1.19/0.95	0.65	0.76
PP6	4.05	1.06	0.89/0.12	0.66	0.79
PP7	3.92	1.10	0.74/0.34	0.74	0.84
PP8	3.95	1.11	0.89/0.03	0.64	0.66
PP9	4.29	0.87	1.14/0.82	0.71	0.80
V: Changing rooms (*α* = 0.96; CR = 0.95; AVE = 0.63)					
V1	4.01	1.16	1.03/0.16	0.75	0.84
V2	3.90	1.16	0.85/0.16	0.68	0.75
V3	3.95	1.16	0.95/0.12	0.74	0.86
V4	3.81	1.31	0.85/0.38	0.71	0.77
V5	4.07	1.20	1.15/0.33	0.60	0.66
V6	3.86	1.19	0.81/0.27	0.70	0.80
V7	3.83	1.16	0.74/0.32	0.73	0.80
V8	4.12	1.08	1.12/0.60	0.70	0.78
V9	3.83	1.21	0.79/0.27	0.75	0.85
V10	4.09	1.03	1.09/0.76	0.79	0.89
V11	3.97	1.12	0.86/0.13	0.76	0.84
V12	4.09	1.12	1.16/0.49	0.74	0.81
PAP: Padel activities programme (*α* = 0.94; CR = 0.94; AVE = 0.66)					
PAP1	3.88	1.14	0.70/0.39	0.78	0.81
PAP2	4.05	1.04	0.85/0.20	0.78	0.82
PAP3	3.68	1.23	0.55/0.65	0.76	0.80
PAP4	3.80	1.26	0.69/0.67	0.70	0.77
PAP5	4.04	1.02	0.91/0.28	0.78	0.84
PAP6	3.96	1.12	0.88/0.03	0.70	0.76
PAP7	3.97	1.08	0.81/0.05	0.78	0.85
PAP8	4.11	1.01	1.08/0.72	0.75	0.82
PAP9	4.24	0.89	1.14/1.10	0.74	0.78
TP: Padel technicians (*α* = 0.97; CR = 0.97; AVE = 0.80)					
TP1	4.21	1.03	1.30/1.22	0.77	0.87
TP2	4.27	0.99	1.43/1.76	0.75	0.89
TP3	4.20	1.06	1.32/1.15	0.77	0.91
TP4	4.26	1.02	1.42/1.63	0.75	0.90
TP5	4.25	0.98	1.36/1.53	0.76	0.92
TP6	4.25	0.98	1.31/1.29	0.74	0.93
TP7	4.27	0.98	1.39/1.60	0.73	0.91
TP8	4.34	0.98	1.55/2.04	0.69	0.83

Note: M, average; SD, standard deviation; Sk, skewness; Ku, kurtosis; λ, factor loading; EFA, exploratory factor analysis; CFA, confirmatory factor analysis; CR, composite reliability; AVE, average variance extracted.

Additionally, complementary measures were analysed to test the reliability and validity of the tool. For the first case, the composite reliability (CR) obtained values higher than 0.70 in all the constructs, its being able to affirm that the items of the manifest variables really measure each of the underlying variables (97). For the second case, the average variance extracted (AVE) was used, which represents the proportion of variance of the construct that can be explained by its indicators, obtaining values higher than the minimum value 0.50 assuming convergent validity, and convergent validity was also analysed using the Fornell-Larcker (98) criterion, where squared correlations were obtained between constructs lower than their respective AVE values (Table 4).

**Table 4:** Discriminant validity.

Constructs	ID	PP	V	PAP	TP
ID	*0*.*59*				
PP	0.26	*0*.*57*			
V	0.35	0.53	*0*.*63*		
PAP	0.26	0.55	0.51	*0*.*65*	
TP	0.40	0.32	0.31	0.59	*0.80*

Note: The values on the diagonal (in italics) correspond to the square root of the shared variance between the constructs and their measures.

## 4 Discussion

The relevance of padel as an emerging sport has attracted attention in the scientific literature in recent years, with several studies focusing on aspects related to the players (performance, psychology, or physiology) as well as certain variables associated with sports management, but the quality of the service has not been adequately addressed. The aim of this study was to adapt and validate a tool to assess the quality perceived by users of padel facilities and services, using a sample of users of this type of facilities and services from the Autonomous Community of Andalusia, a region located in the south of Spain.

Aparicio-Sarmiento et al. (22) focused their study on the analysis of the satisfaction of padel users in a sample of 36 participants, using the EPOD tool (53), originally designed for the evaluation of user satisfaction in sports organisations. However, the items used in the study have a shorter wording than the original version without describing the adaptation process used, without providing information on the psychometric properties, as well as using the construct satisfaction and not perceived quality. Perceived quality implies according to Bitner and Hubbert (99) a consumer's impression of the relative superiority or inferiority of an organisation and its services, whereas satisfaction implies a post-consumer (100) or post-purchase (101) response or evaluation, hence quality is considered an antecedent of satisfaction (62) and satisfaction an antecedent of behavioural intention (102). This is in line with Baena-Arroyo et al.'s (35) assertion that perceived quality is a first step to customer loyalty, while other variables such as satisfaction are determinants of consumer behaviour (87).

Perceived quality is one of the variables that have received most attention in the sport industry. In the present study, with the intention of filling a gap in the literature, a review of tools designed for the evaluation of perceived quality in different contexts within sport services was carried out, considering the use of the CECASDEP tool for adaptation to the context of padel. Its internal structure integrates 3 of the most commonly used dimensions in the literature (see Table 1), namely personnel (referring to the human resources and workers of the specific service), programmes (referring to the contents, services and activities of padel), and facilities (referring to the global installation that integrates the different sports spaces necessary for the development of the sport activity).

These 3 dimensions include a specific dimension for the changing room and a dimension for the playing space itself (padel courts). In the case of the changing room, it is a space necessary at a functional level (e.g., change of clothes, hygiene, safety through lockers), and at the level of user experience (pleasant atmosphere, cleanliness, additional or complementary equipment, etc.), including in both cases adaptability and accessibility for people with functional diversity. However, this dimension is only used in the SQAS tool (40) and in the adaptation made by Yu et al. (80), although there are some tools that address this space, although not in such a specific way. This is the case of the QUESC scale (38) which includes the dimension “ambiance” with 7 items, 3 of them referring to changing rooms (comfortable temperature, warm changing room, and cleanliness); the HATSQ scale (70) includes the dimensions “ambience and accessibility” where 2 items refer to changing rooms (changing rooms and hygiene in the shower area); the NEPTUNO scale (73) included a specific dimension for cleanliness where they dedicated 1 item to ask about the cleanliness of the toilets; the EPOD scale used by Aparicio-Sarmiento et al. (22) for the satisfaction of padel clubs refers to the changing room using only 1 item to assess cleanliness. As for the specific dimension on padel courts, no tools have been found that address this specific sports space, so the present study adapted the original dimension called “sports spaces” as padel courts are considered a determining factor in the perception of service quality, this criterion contributing to a better positioning in a competitive market. The adaptation process led to the elimination of 1 item that lacked the possibility of adaptation due to lack of application to the specific context (“the acoustics of the sports spaces are good”), and the remaining items addressed aspects related to dimensions, lighting, playing surface, net, maintenance, cleanliness, orientation and safety.

The results obtained in this study show adequate psychometric properties of the CECASDEP tool adapted to the context of padel. Given the importance of the analysis of perceived quality in the sports sector, it is crucial to have tools that comply with a rigorous methodology and that allow a valid and reliable assessment to be carried out. In this sense, the QPadel tool has an internal structure of 5 dimensions and 48 items that allow the evaluation of the perceived quality of users of padel facilities and services, and also represents a novel contribution to the literature as it is a validated tool for a context in which there is a significant gap. Additionally, it has a great potential for adaptability to different contexts related to racket and padel sports, and consequently, to different practical realities by providing an important aid to managers of padel facilities for the analysis of the perceived quality of their customers and users, facilitating the establishment of strategies to generate a service that allows a better adaptation to the needs and interests.

### 4.1 Practical implications

The QPadel tool, adapted and validated for the present study, makes an important contribution to the literature by filling an existing gap and has a broad applicability by providing the possibility to assess the quality perceived by users. Therefore, and considering the relevance of providing a superior service for the survival of a service organisation (103), the assessment of perceived quality will facilitate decision-making and the use of available resources for the improvement of quality, and consequently, satisfaction and loyalty.

The results obtained confirm that the dimensions used are relevant in the context of padel, obtaining adequate psychometric properties in the different analyses developed. This is especially remarkable in a context that has experienced a high boom and growth, as it provides organisations with a reliable and valid tool.

Focusing on managers, the results of this study have important implications considering the relevance of the information provided by each of the dimensions, impacting positively on the development of competitive strategies by allowing the identification of areas for improvement in aspects related to these dimensions, such as including improvements in infrastructure, providing specific training for staff, or the creation of programmes of activities and/or events tailored to the needs of users. Therefore, we want to encourage sport managers of padel-related facilities to work with a validated tool that facilitates the establishment of quality standards within the padel industry. It can also serve as a starting point for a benchmarking process that promotes healthy competition and an overall increase in the levels of quality offered to users.

We believe that the implementation of the findings of this study can help to achieve a significant improvement in the management and operation of padel sport facilities, increasing user satisfaction and loyalty and strengthening the competitive position of organisations.

### 4.2 Limitations and future directions

Future studies need to be aware of the limitations found in the present research. The volume of users in padel clubs is not comparable to that of other sporting activities and the frequency of use is also lower, so the sample collection is not an easy task and could not be carried out in a short space of time. Despite the support of the Andalusian Padel Federation, which facilitated the distribution of the tool throughout the Autonomous Community of Andalusia, we were not able to make a specific selection of the participating padel clubs and this provided an imbalance between public and private clubs (types of facilities). Another imbalance was obtained in the gender variable, where the sample of women was much lower than that of men and prevented us from going deeper into specific perceptions and analysing the possible existence of differences. In relation to the frequency of use (weekly practice), we consider it necessary to increase the participants of users who practice padel between 3 and 4 times a week by adding a specific question that allows us to identify whether they practice in the same sports facility or not, so that we can identify whether there is any reason other than proximity (convenience of service) that is associated with the perceived quality, and also, consider the variables age and level of play to check for possible discrepancies in perceived quality, all aimed at better management of both the facilities and the services offered.

## 5 Discussion

The results obtained in this study show adequate psychometric properties of the QPadel assessment tool, which comes from the adaptation of another perceived quality evaluation tool after an indepth review of the literature. Specifically, QPadel responds to the consideration of the three dimensions most commonly used in the literature (facilities, program and staff). These dimensions include items that encompass user attention, location and external equipment (facilities), characteristics and expectations (programme), and, finally, content and interaction (staff). In addition, this tool dedicates a specific dimension to the sports space where the activity takes place, including items on comfort and functionality, and last of all, in the case of the changing room dimension, it includes items on environmental elements and comfort.

Given the importance of the analysis of perceived quality in the sports sector, it is crucial to have tools that comply with a rigorous methodology and that allow a valid and reliable assessment to be carried out. In this sense, the QPadel tool has an internal structure of 5 dimensions and 48 items that allow the evaluation of the perceived quality of users of padel facilities and services, and also represents a novel contribution to the literature as it is a validated tool for a context in which there is a significant gap. Additionally, it provides important help to managers of padel facilities for the analysis of the perceived quality of their customers and users, facilitating the establishment of strategies to generate a service that allows a better adaptation to the needs and interests.

## Data Availability

The raw data supporting the conclusions of this article will be made available by the authors, without undue reservation.

## Appendix A. Survey items

*Sports facilities*

ID1: The sports facility is well located

ID2: I find it easy to get to the sports facility

ID3: I find the green areas adequate.

ID4: I find it easy to park when I go to the sports facility.

ID5: The space in the reception area is adequate for my attention.

ID6: The control of users at the reception is adequate.

ID7: The means of transmitting suggestions and/or complaints are adequate.

ID8: In the event of a problem or complaint, I know who to contact.

ID9: When I have a problem, the willingness to help me is good.

ID10: The treatment I receive is friendly.

*Padel courts*

PP1: The external dimensions of the courts where I play are adequate.

PP2: The lighting of the courts is appropriate.

PP3: The playing surface is in perfect condition.

PP4: The net is in good condition and undamaged.

PP5: The net is taut and suitable for play.

PP6: The maintenance of the glass/walls seems to me to be correct.

PP7: The cleanliness of the pitch seems to me to be correct.

PP8: The orientation of the courts is correct.

PP9: The courts are safe for me.

*Changing rooms*

V1: The dimensions of the changing rooms are adequate for my comfort.

V2: The provision of benches is sufficient for my comfort.

V3: The size of the shower area is appropriate.

V4: The lockers offer me security.

V5: The toilets are located outside the shower area.

V6: The ventilation of the toilets is adequate.

V7: The floor is non-slip and safe for me.

V8: The temperature of the water in the showers is comfortable.

V9: The ventilation of the changing rooms is adequate.

V10: I find the lighting to be correct.

V11: The temperature is comfortable.

V12: The cleanliness is adequate.

*Padel activities programme*

PAP1: There is a wide range of activities on offer.

PAP2: It was easy to obtain information about the activities on offer.

PAP3: Activities are changed frequently during the season.

PAP4: Occasional activities take place during the season (tournaments, pull, etc.).

PAP5: The activity in which I participate meets my expectations.

PAP6: The price of the activity is appropriate to the service I receive.

PAP7: The weekly distribution (frequency) of the activities is appropriate.

PAP8: The timetable of the activity is appropriate.

PAP9: The duration of the activity is appropriate.

*Padel technicians*

TP1: There is good communication between the users and the technician.

TP2: The treatment with the technician is pleasant.

TP3: The classes are well organised.

TP4: The coach takes care to adapt the activity to the level of the users.

TP5: The technician distributes the time available appropriately.

TP6: The technician makes good use of the materials at his disposal.

TP7: The technician's involvement during the activity is adequate.

TP8: The technician is capable of carrying out the activity.
